# Effect of the COVID-19 pandemic on complications and mortality of patients with cardiac surgery

**DOI:** 10.1186/s13019-021-01744-z

**Published:** 2021-12-31

**Authors:** Xue Wang, Heng Gao, Zhanqin Zhang, Chao Deng, Yang Yan, Tao Shi

**Affiliations:** 1 Department of Cardiovascular Surgery, First Affiliated Hospital of Xi’an Jiaotong University, No. 277 Yanta West Road, Xi’an, 710061 People’s Republic of China; 2 Department of Emergency Internal Medicine, Shaanxi Provincial People’s Hospital, 256 Youyi West Road, Xi’an, 710068 People’s Republic of China

**Keywords:** COVID-19 pandemic, Cardiovascular disease, Cardiac function, Complications, Prognosis

## Abstract

**Objectives:**

The purpose of this study was to assess changes in cardiovascular disease severity, types, postoperative complications and prognosis during the COVID-19 pandemic and to explore possible influencing factors.

**Methods:**

A total of 422 patients were enrolled in this study, and hospitalization and short-term follow-up data were retained. The patient population included 273 men and 149 women. Patients had a median (IQR) age of 54 (45–62) years and were divided into an observation group (130) and a control group (292), primarily according to severity of disease, disease types, baseline indexes, biochemical indexes, cardiac function indexes, complications and prognosis.

**Results:**

During the COVID-19 pandemic, compared with the same period last year, there was a significant increase in patients with aortic dissection (27.69% vs 5.82%), a significant decrease in patients with valvular heart disease (43.08% vs 66.78%), and significantly increased emergency admission (50.00% vs 21.23%) and severity (54.62% vs 27.40%). Family company (76.37% vs 64.62%) was decreased, EuroSCORE [6.5 (2–9) vs 2 (0–5)] score, Pro-BNP [857.50 (241.00–2222.50) vs 542.40 (113.45–1776.75)] ng/L, six months mortality rate (18.46% vs 8.90%), and postoperative complications, including infected patients, atelectasis, pulmonary edema, and so on were increased, with longer length of stay in the ICU and hospital in COVID-19 pandemic. Survival analysis curve further demonstrated that it had an impact on the deaths of patients during the COVID-19 pandemic period. Through ROC analysis of the death factors of patients, it was concluded that Family company affected the death of patients, and the area under the curve was 0.654 (*P* < 0.05).

**Conclusions:**

In this study, we found that the admission rate of critically ill patients with cardiovascular disease, complications of cardiac surgery, and short-term mortality of patients all exhibited a short-term increase, family company may be a risk factors for short-term mortality, that may be related to public pressure caused by the COVID-19 pandemic.

## Introduction

In December 2019, coronavirus disease 2019 (COVID-19) was identified in Wuhan, China; this disease is caused by severe acute respiratory syndrome coronavirus 2 (SARS-CoV-2) [1]. As COVID-19 infections continue to increase worldwide, a large number of morbidities and mortalities are being reported. By 16 March 2020, China reported 44,672 cases of infection due to COVID-19. A total of 10.5% of those had cardiovascular disorders, 7.3% had diabetes, and 6.0% had hypertension, suggesting that this virus severely affects patients who have heart conditions. Research has shown that there has been a decrease in daily hospital admissions, such as due to angiocardiopathy, during the pandemic. According to a previous report, a 58% increase in out-of-hospital cardiac arrest was strongly associated with cumulative COVID-19 incidence, which may be associated with the avoidance of medical care due to social distancing and concerns of contracting COVID-19, as well as social stress and bad habits [2–4]. Acute cardiovascular events are associated with many factors, including primary disease, predisposing factors, and unreasonable management, which may be a trigger for aggravating the occurrence of acute cardiovascular events, affecting postoperative recovery from cardiovascular diseases. During the outbreak of COVID-19, the number of prehospital cardiovascular deaths increased [3], but in the hospital setting, whether the patient's outcome was affected by active surgery or good and complete care has not been studied.

Although it is clear that COVID-19 primarily impairs the respiratory system, the underlying cardiovascular performance of non-COVID-19 patients in the context of the epidemic is not well understood and has not been studied. Therefore, we enrolled 422 patients and retained hospitalization and short-term follow-up data. During the COVID-19 pandemic, compared with the same period last year, we assessed the severity, complications, and prognosis of patients undergoing cardiac surgery by analyzing and collecting disease type, pathogenesis characteristics, disease development indicators, and prognostic indicators to explore potential influencing factors. These findings provide the basis of targeted interventions for cardiovascular disease in China during the COVID-19 outbreak.

## Methods

### Study design and participants

This descriptive, cross-sectional study was conducted in China. All patients were recruited from cardiovascular surgery at the First Affiliated Hospital, Medical School of Xi’an Jiaotong University, Xi’an. We enrolled 422 patients, including 273 men and 149 women. The patients had a median (IQR) age of 54 (45–62) years, and they were divided into an observation group and a control group. Patients in the observation group were admitted for cardiovascular surgery between January 23 and April 8, 2020, during the COVID-19 pandemic. Patients in the control group were admitted for cardiovascular surgery between January 23 and April 8, 2019, during the same period last year.

We applied 2 inclusion criteria: (1) all patients diagnosed with cardiovascular disease according to guidance and gold standard; (2) all patients underwent a full set of routine laboratory tests, including compete blood count, urinalysis, blood biochemistry, blood gas analysis, blood coagulation function and echocardiography. Patients who did not meet the above inclusion criteria were excluded from the study.

### Procedures

We collected data on type of disease, baseline, clinical, biochemical, cardiac function, intraoperative, postoperative complications and outcome from all 422 patients. Outcome measures were obtained from the patients’ medical records. We followed up with the patients’ symptoms 1–6 months after surgery through outpatient and telephone visits. To collect data as comprehensively as possible, we used a combination of chart review and, if necessary, communicating with the attending doctors and other medical workers to fill in missing data.

### Statistical analysis

Categorical variables are reported as numbers and percentages, and continuous variables are reported as medians with interquartile ranges (IQRs). Proportions were compared by the chi-square test, and continuous variables were compared by the Mann–Whitney test. For laboratory results, we also assessed whether measurements were outside the normal range. Survival rate according to the Kaplan-Meier technique, and event-free curves were compared with the use of the log-rank test. ROC is used to analyze the influence intensity of major event factors, All statistical analyses were performed using IBM Statistics 26. A two-tailed value of *P* < 0.05 was considered statistically significant.

## Results

### Comparison of the proportions of different diseases between the two groups

Compared to the observation group (17/292), the control group (36/130) had a significantly decreasing trend for aortic dissection (27.69% vs 5.82%) (*P* < 0.01). The total number of patients receiving V-A ECMO treatment in the observation group (2/130) was 1.54%, while the total number of patients in the control group was 1.03% (3/292), which was not statistically different (*P* > 0.05). A total of 43.08% of patients (56/130) had valvular heart disease in the observation group, which was significantly lower than the observation of 66.78% of patients (195/292) with valvular heart disease in the control group (*P* < 0.01). A total of 1.54% of patients (2/130) had constrictive pericarditis in the observation group, similar to 0% of patients (0/292) with constrictive pericarditis in the control group (*P* > 0.05). Moreover, a total of 13.85% of patients (18/130) had congenital heart disease in the observation group, similar to 15.07% of patients (44/292) with congenital heart disease in the control group (*P* > 0.05). In addition, compared with the observation group of patients with coronary heart disease (16/130), the control group (33/292) had a similar result (12.31% vs 11.30%) (*P* > 0.05) (Table 1).

**Table 1:** Comparison of the proportions of different diseases between the two groups

Type of disease	All patients (n = 422)	Control group (n = 292)	Observation group (n = 130)	*P*-value
Aortic dissection	53	17	36	0.000
V-A ECMO	5	3	2	0.654
Valvular heart disease	251	195	56	0.000
Constrictive pericarditis	2	0	2	0.344
Congenital heart disease	62	44	18	0.743
Coronary heart disease	49	33	16	0.766

### Comparison of baseline, biochemical and cardiac function indexes between the two groups

During the COVID-19 pandemic, compared with the same period last year, we found that the proportion of patients from Shaanxi Province (81.77% vs 62.33%), smoking (44.62% vs 34.25%), alcoholism (14.62% vs 5.48%), arteriography (60.77 vs 40.41), EuroSCORE [6.5 (2–9) vs. 2 (0–5)] score, emergency admission (50.00% vs 21.23%), severe patients (54.62$% vs 27.40%), leucocyte count [6.59 (4.95–9.62) vs 6.14 (4.86–8.08)]10^9^/L and Pro-BNP [857.50 (241.00–2222.50) vs 542.40 (113.45–1776.75)] ng/L were increased (*P* < 0.05). In contrast, albumin [37.15 (34.83–40.03) vs 40.50 (37.50–43.88)] g/L, partial pressure of oxygen [80.30 (71.15–89.38) vs 85.15 (74.60–93.55)] mmHg and Family company (76.37% vs 64.62%) were significantly lower (*P* < 0.01). However, other indicators of coagulation function were not significantly different (*P* > 0.05) (Table 2).

**Table 2:** Comparison of preoperative baseline, biochemical and cardiac function indexes between the two groups

Characteristics	Control group (n = 292)	Observation group (n = 130)	*P*-value
Age, years	55 (44–62)	54 (47–61)	0.935
Countryside (%)	187 (64.04)	82 (63.08)	0.849
Men (%)c	184 (63.01)	89 (68.46)	0.280
Shaanxi Province (%)	182 (62.33)	105 (81.77)	0.000
Smoking (%)	100 (34.25)	58 (44.62)	0.042
Alcoholism (%)	16 (5.48)	19 (14.62)	0.002
Distance (km)	132 (35.5–237)	142 (42.25–237.5)	0.778
Family company (%)	223 (76.37)	84 (64.62)	0.012
BMI (kg/m^2^)	23.15 (20.43–25.70)	24.14 (21.25–25.95)	0.130
History of hypertension (%)	77 (26.37)	43 (33.08)	0.159
History of diabetes (%)	32 (10.96)	10 (7.69)	0.301
History of COPD (%)	9 (3.08)	3 (2.31)	0.659
History of hyperlipidemia (%)	40 (13.70)	27 (20.77)	0.067
PAH (%)	59 (20.21)	18 (13.85)	0.118
Arteriography (%)	118 (40.41)	79 (60.77)	0.000
EuroSCORE (score)	2 (0–5)	6.5 (2–9)	0.000
Emergency admission (%)	62 (21.23)	65 (50.00)	0.000
Severe patients (%)	80 (27.40)	71 (54.62)	0.000
Disease time, years	3.96 ± 10.19	2.54 ± 6.25	0.144
HGB (g/L)	132 (122–142)	134 (121–143)	0.881
Eosinophil count (10^9^/L)	0.07 (0.04–0.14)	0.06 (0.02–0.14)	0.323
Leucocyte count (10^9^/L)	6.14 (4.86–8.08)	6.59 (4.95–9.62)	0.029
Lymphocyte count (10^9^/L)	1.48 (1.04–1.97)	1.33 (0.96–1.72)	0.057
ALB (g/L)	40.50 (37.50–43.88)	37.15 (34.83–40.03)	0.000
Globulin (U/ml)	24.50 (21.03–27.90)	25.10 (22.08–27.93)	0.216
Partial pressure of oxygen (mmHg)	85.15 (74.60–93.55)	80.30 (71.15–89.38)	0.007
Pro-BNP (ng/L)	542.40 (113.45–1776.75)	857.50 (241.00–2222.50)	0.018
LVEF (%)	59 (50–64)	59 (51–63)	0.828

### Comparison of postoperative complications and outcome indicators between the two groups

**Fig. 1:**
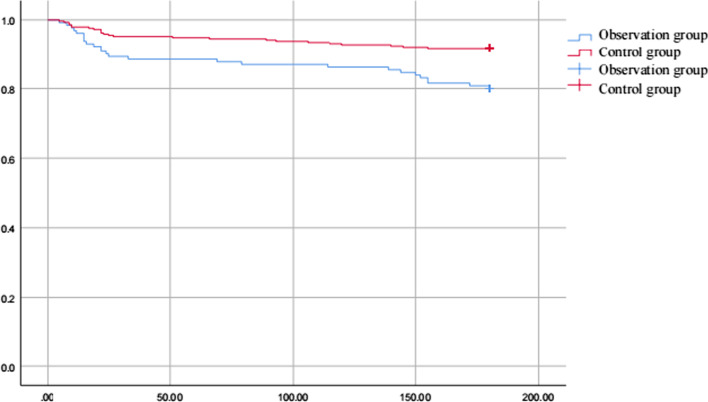
Cumulative survival in each group

**Fig. 2:**
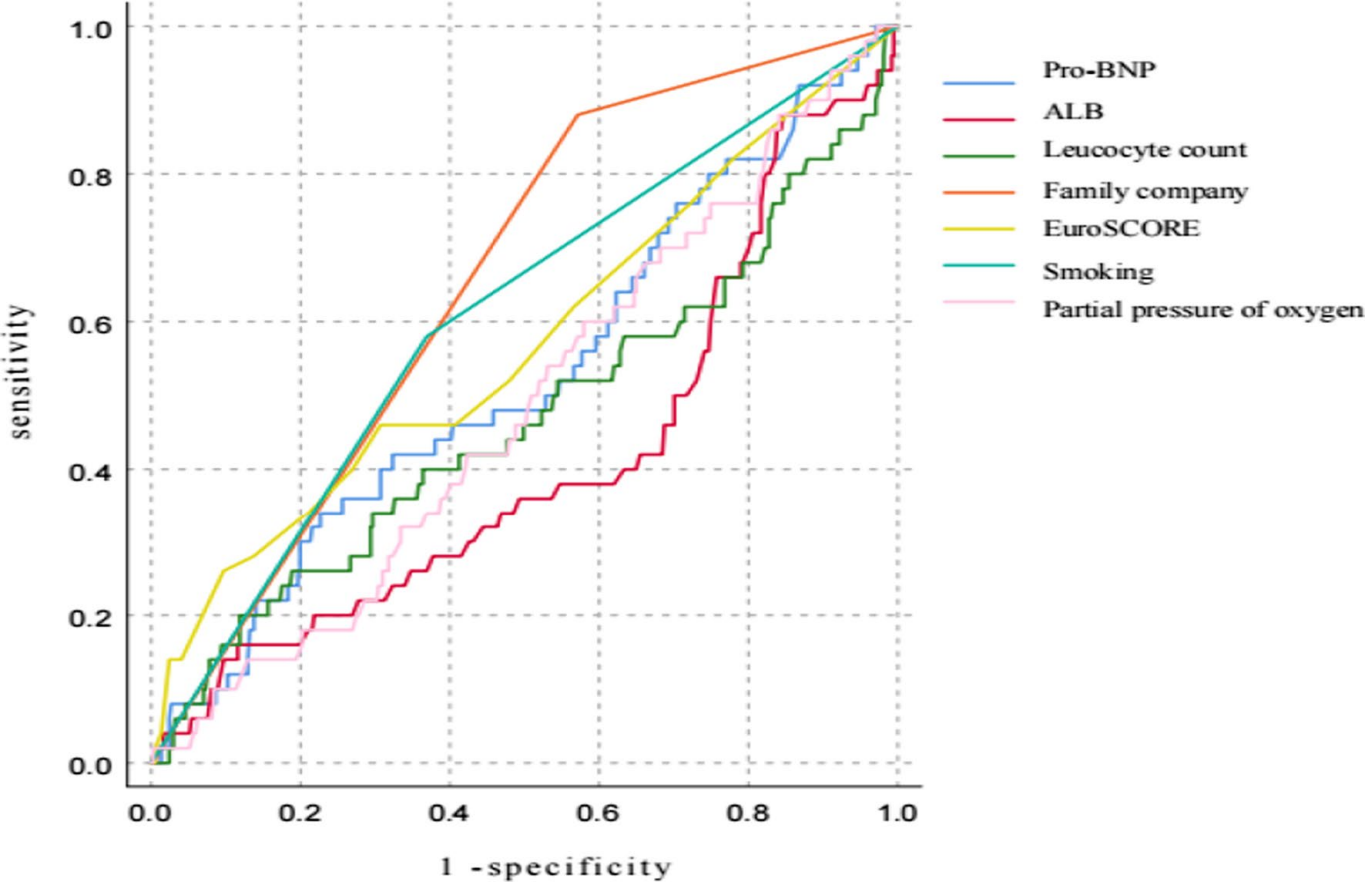
ROC curve of a single index of Pro-BNP, ALB, Leucocyte count, Family company, Smoking, EuroSCORE, Partial pressure of oxygen for predicting deaths in patients. ROC, receiver operating characteristics

**Fig. 3:**
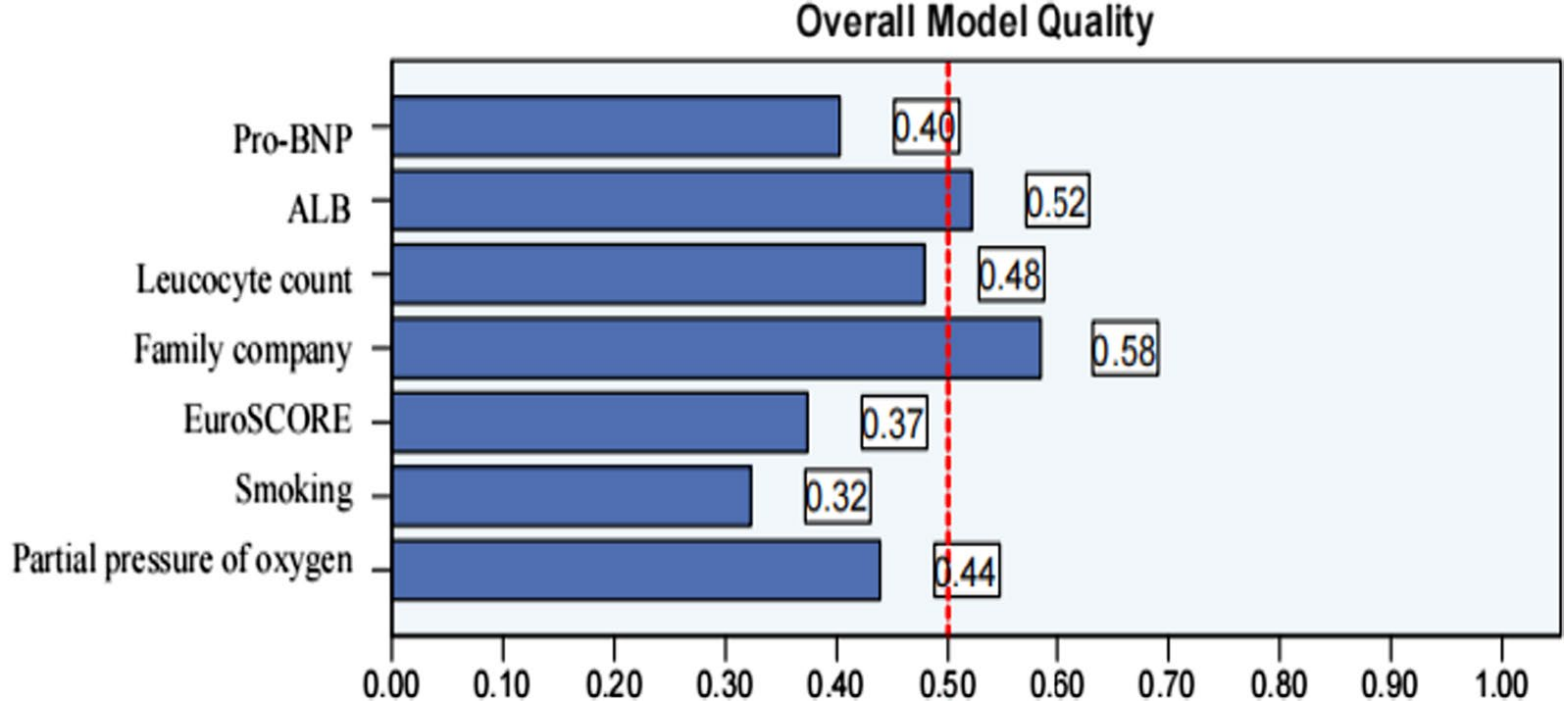
Overall quality of the ROC analysis, good models have values above 0.5, A value less than 0.5 indicates that the model is not better than Independent prediction

Ventilator assistance time [1 (1–2) vs 1 (0.5–1)] days, length of stay in the ICU [4 (2–6) vs 2 (1–3)] days and length of stay in the hospital [17 (13–23) vs 12 (10–17)] in the observation group were significantly higher than those in the control group (*P* < 0.01). Infected patients [(20.77%, 27 out of 130)vs (4.11%, 12 out of 292)], atelectasis [(27.69%, 36 out of 130) vs (7.19%, 21 out of 292)], pulmonary edema [(10.00%, 13 out of 130) vs (1.03%, 3 out of 292)], gastrointestinal dysfunction [(21.54%, 28 out of 130) vs (3.08%, 9 out of 292)], atrial arrhythmia [(9.23%, 12 out of 130) vs (1.37%, 4 out of 292)], secondary tracheal intubation [(12.31%, 16 out of 130) vs (2.74%, 8 out of 292)], acute renal injury [(10.00%, 13 out of 130) vs (2.40%, 7 out of 292)], coagulation disorders [(1.62%, 6 out of 130) vs (0.34%, 1 out of 292)], total blood transfusion [1700 (920–3060) vs 600 (400–1000)], total mediastinal drainage [98 (609–2481) vs 625 (372–1114)] and six months mortality rate [(18.46%, 24 out of 130) vs (8.90%, 26 out of 292)] of the observation group were significantly higher than those in the control group (*P* < 0.01). However, there was no difference between the observation and control groups in terms of ventricular arrhythmia, acute liver injury, stroke, delirium, secondary hemostasis, or 30-day mortality rate (*P* > 0.05) (Table 3). Survival analysis curve further demonstrated that it had an impact on the deaths of patients during the COVID-19 pandemic period (Fig. 1, log-rank x^2^ = 18.68, *P* < 0.05). Through ROC analysis of the death factors of patients, it was concluded that Family company affected the death of patients, and the area under the curve was 0.654 (Figs. 2, 3, *P* < 0.05).

**Table 3:** Comparison of postoperative complications and outcome indicators between the two groups

Characteristics	Control group (n = 292)	Observation group (n = 130)	*P-*value
Ventilator assistance time (day)	1 (0.5–1)	1 (1–2)	0.000
Length of stay in the ICU (day)	2 (1–3)	4 (2–6)	0.000
Length of stay in the hospital (day)	12 (10–17)	17 (13–23)	0.000
*Postoperative complications*			
Infected person (%)	12 (4.11)	27 (20.77)	0.000
Atelectasis (%)	21 (7.19)	36 (27.69)	0.000
Pulmonary edema (%)	3 (1.03)	13 (10.00)	0.000
Gastrointestinal dysfunction (%)	9 (3.08)	28 (21.54)	0.000
Ventricular arrhythmia (%)	10 (3.42)	10 (7.69)	0.057
Atrial arrhythmia (%)	4 (1.37)	12 (9.23)	0.000
Secondary tracheal intubation (%)	8 (2.74)	16 (12.31)	0.000
Acute kidney injury (%)	7 (2.40)	13 (10.00)	0.001
Acute liver injury (%)	4 (1.37)	4 (3.08)	0.235
Coagulation disorders (%)	1 (0.34)	6 (4.62)	0.002
Stroke (%)	7 (2.40)	7 (5.38)	0.114
Delirium (%)	7 (2.40)	6 (4.62)	0.223
Secondary hemostasis (%)	5 (1.71)	2 (1.54)	0.897
Total blood transfusion (ml)	600 (400–1000)	1700 (920–3060)	0.000
Total mediastinal drainage (ml)	625 (372–1114)	980 (609–2481)	0.000
30-day mortality rate (%)	18 (6.16)	14 (10.77)	0.099
6 months mortality rate (%)	26 (8.90)	24 (18.46)	0.005

### Comparison of the two groups with different grades in EuroSCORE

The observation group exhibited significantly higher EuroSCORE > 9 score [(20.77%, 27 out of 130) vs (7.19%, 21 out of 292)] compared to those in the control group (*P* < 0.01). Furthermore, patients with EuroSCORE 7–9 score [(30.00%, 39 out of 130) vs (47.26%, 138 out of 292)] in the observation group were significantly reduced compared to those in the control group (*P* < 0.01). Finally, patients with EuroSCORE 1–3 score and 4–6 score in the observation group was similar to the control group, with no significant difference between the two groups in this score range (*P* > 0.05) (Tables 4).

**Table 4:** Comparison of the two groups with different scores in EuroSCORE

Characteristics	Control group (n = 292)	Observation group (n = 130)	*P*-value
EuroSCORE 1–3 score	77 (26.37)	40 (30.77)	0.351
EuroSCORE 4–6 score	56 (19.18)	24 (18.46)	0.862
EuroSCORE 7–9 score	138 (47.26)	39 (30.00)	0.001
EuroSCORE > 9 score	21 (7.19)	27 (20.77)	0.000

### ROC analysis of death

Through ROC analysis of the death factors of patients, it was concluded that Family company affected the death of patients. The area under the ROC curve (AUC) of Family company was 0.654 (HR 0.036, 95% CI, 0.583 to 0.725; *P* < 0.05); the AUC of ALB was 0.411 (HR 0.049, 95% CI, 0.323 to 0.499; *P* < 0.05); the AUC of Smoking was 0.605 (HR 0.043, 95% CI, 0.521 to 0.689; *P* < 0.05) (Table 5).

**Table 5:** ROC analysis of different factors affecting death

Variables	AUC	SE	95% CI	*P* Value
Pro-BNP (ng/L)	0.527	0.045	0.439–0.615	0.551
ALB (g/L)	0.411	0.049	0.323–0.499	0.048
Leucocyte count (109/L)	0.469	0.049	0.374–0.564	0.523
Family company (%)	0.654	0.036	0.583–0.725	0.000
Smoking (%)	0.605	0.043	0.521–0.689	0.014
Partial pressure of oxygen (mmHg)	0.485	0.042	0.403–0.567	0.721
EuroSCORE (score)	0.571	0.047	0.479–0.663	0.131

## Discussion

We observed the characteristics, complications, and prognosis of patients with cardiovascular disease during the COVID-19 pandemic and compared them to the same period last year. We observed that the number of cardiac surgery patients significantly decreased compared to last year, and the proportion of diseases was significantly different. The proportion of patients with aortic dissection significantly increased compared to last year, and patients with valvular heart disease significantly decreased compared to last year, while other diseases were similar in proportion, that may be related to the willingness of patients to seek medical care during the COVID-19 pandemic period.

By analyzing the baseline, biochemical, and cardiac function indexes in the two groups, comparing this year to the same period last year, the proportion of patients, from Shaanxi Province, emergency admission, severe patients, EuroSCORE score and Pro-BNP significantly increased during the COVID-19 pandemic. However, Family company during the COVID-19 pandemic period were significantly lower than those in the same period last year.

Further, by analyzing indexes of postoperative complications and prognostic, we observed that compared to the same period last year, Ventilator assistance time in days, length of stay in the ICU in days and length of stay in the hospital during the COVID-19 pandemic period were significantly higher than those in the same period last year. Infected patients, atelectasis, pulmonary edema, gastrointestinal dysfunction, atrial arrhythmia, secondary tracheal intubation, acute renal injury, coagulation disorders, total mediastinal drainage and six months mortality rate during the COVID-19 pandemic period were significantly higher than those in the same period last year. Based on the severity of EuroSCORE, patients with EuroSCORE > 9 score during the COVID-19 pandemic period were significantly higher than those in the same period last year. Furthermore, patients with EuroSCORE 7–9 score during the COVID-19 pandemic period were significantly reduced compared to those in the same period last year. Survival analysis curve further demonstrated that it had an impact on the deaths of patients during the COVID-19 pandemic period. Through ROC analysis of the death factors of patients, it was concluded that Family company affected the death of patients.

In this study, we found that compared with the same period of last year, patients with cardiac surgery were more serious, the number of patients with emergency and emergency surgery increased, postoperative complications significantly increased, and prognosis was poor.

Many studies have shown that acute cardiovascular disease increases within hours to days after psychological triggers, including anger, depression, anxiety, and stress [5–10]. In this study, Family company during the COVID-19 pandemic period was significantly lower than those in the same period last year. So we guess: during the COVID-19 pandemic period, Lack of family company made patients psychological change, Many studies also have shown the daily increase in COVID-19 patients and patients who die have made people anxious, depressed and experience great pressure [11–13], especially in patients with cardiovascular disease, where a series of methods to relieve bad emotions, such as anxiety, followed, such as smoking, drinking, overeating, and loss of temper [14–17]. As a result of the progress and deterioration of the disease, the lack of timely treatment and changes in the attitude of seeking medical treatment, if the condition is not serious, patients do not go to the hospital to see a doctor [18, 19], which may affect the occurrence of complications and prognosis in patients with cardiac surgery.

Therefore, during future outbreaks of the epidemic, it is necessary to establish a fast track as soon as possible, not only in terms of the hospital but also in terms of the patient's time and methods to reach the hospital [20]. It is important to establish a cardiovascular disease database and to regularly follow-up with patients to urge them to seek medical treatment in time and develop decompression and good eating habits during the outbreak, family members of patients who are quarantined in situ due to the COVID-19 pandemic should actively contact their family members and entrust their friends to accompany the patients to reduce their anxiety, in order to reduce the occurrence of acute cardiovascular events and postoperative complications during the epidemic and improve the survival rate. This study has few cases, short follow-up time, is a single-center study, and no direct study on the relationship between stress and prognosis of patients undergoing cardiovascular surgery was performed. Further analysis of possible influencing factors may provide a theoretical basis for what measures we take to benefit patients in the event of major public health events.

Finally, seasonal effects due to daylight savings time have been shown to increase the risk of CVD events (32), and we cannot rule out the potential influence of seasonality on our results. However, confounding is of minimal concern given the short event windows chosen for the present analysis.

## Conclusion

During the COVID-19 pandemic period, different types of cardiovascular surgery patients were admitted to hospitals, The pandemic increased postoperative complications after heart surgery, and increased the short-term mortality of patients. The presence or absence of family members affected short-term mortality of patients, which may be related to the public pressure caused by the COVID-19 pandemic. which may be attributed to public pressure from the COVID-19 pandemic. Further research is needed to understand the association of anxiety, depression, stress, etc., with acute cardiovascular disease and other possible influencing factors.

### Perspectives

#### Competency in medical knowledge

The pandemic has briefly increased the admission rate of acute cardiovascular disease, reduced the admission rate of chronic disease, increased postoperative complications after heart surgery, and increased the short-term mortality of patients, which may be attributed to public pressure from the COVID-19 pandemic.

#### Translational outlook

Further research is needed to understand the association of anxiety, depression, stress, etc., with acute cardiovascular disease and other possible influencing factors.

## Data Availability

All data generated or analysed during the current study are available from the corresponding author on reasonable request.

## Central illustration

Flowchart describing the process of patient selection using the latest diagnostic criteria for various cardiovascular diseases and patients who underwent surgery. Based on this, a total of 430 consecutive patients with cardiovascular diseases were enrolled from January 23, 2019, to April 8, 2019, and from January 23, 2020, to April 8, 2020. Eight patients were excluded due to loss of contact with patients and incomplete data. Finally, data from 422 patients were extracted and divided into two groups: the observation group patients were admitted to cardiovascular surgery between January 23 and April 8, 2020, during the COVID-19 pandemic, and the control group of patients was admitted to cardiovascular surgery between January 23 and April 8, 2019, the same period last year. We collected data on the type of disease, baseline, clinical, biochemical, cardiac function, intraoperative, postoperative complications and outcome for all 422 patients. Clinical outcomes were followed up to 1–6 months after surgery, including outpatient and telephone follow-up. Data were collected as comprehensively as possible through a combination of chart review and, when necessary, communication with attending doctors and other medical workers to fill in the missing data. All data were separately extracted by 2 authors.

## Abbreviations

IQR: Interquartile range
ICU: Intensive care unit
ROC: Receiver operator characteristic curve
V-A ECMO: Venous-artery extracorporeal membrane oxygenation
ALB: Serum albumin
BMI: Body mass index
COPD: Chronic obstructive pulmonary disease
PAH: Pulmonary arterial hypertension
HGB: Haemoglobin
LVEF: Left ventricular ejection fraction

## References

[1] Choudhary S, Sreenivasulu K (2021). Role of genetic variants and gene expression in the susceptibility and severity of COVID-19. Ann Lab Med.

[2] De Filippo O, D’Ascenzo F, Angelini F (2020). Reduced rate of hospital admissions for ACS during Covid-19 outbreak in Northern Italy. New Engl J Med.

[3] Baldi E, Sechi GM, Mare C (2020). Out-of-hospital cardiac arrest during the Covid-19 outbreak in Italy. New Engl J Med.

[4] Brant LCC, Nascimento BR (2020). Excess of cardiovascular deaths during the COVID-19 pandemic in Brazilian capital cities. Heart.

[5] Schneider LH, Hadjistavropoulos HD, Dear BF (2020). Efficacy of internet-delivered cognitive behavioural therapy following an acute coronary event: a randomized controlled trial. Internet Interv.

[6] Giannitsi S, Tsinivizov P, Poulimenos LE (2020). [Case report] stress induced (Takotsubo) cardiomyopathy triggered by the COVID-19 pandemic. Exp Ther Med.

[7] Masters KS, Shaffer JA, Vagnini KM (2020). The impact of psychological functioning on cardiovascular disease. Curr Atheroscler Rep.

[8] Howard R, Kuhn L (2020). Physical health assessment and cardiometabolic monitoring practices across three adult mental health inpatient units—a retrospective cohort study. Int J Ment Health Nurs.

[9] Kivimäki M, Steptoe A (2018). Effects of stress on the development and progression of cardiovascular disease. Nat Rev Cardiol.

[10] Smyth A, O'Donnell M, Lamelas P (2016). Physical activity and anger or emotional upset as triggers of acute myocardial infarction: the interheart study. Circulation.

[11] Hyland P, Shevlin M. Anxiety and depression in the Republic of Ireland during the COVID-19 pandemic*.* 2020. 10.1111/acps.13219 32716520

[12] van der Velden PG, Contino C, Das M (2020). Anxiety and depression symptoms, and lack of emotional support among the general population before and during the COVID-19 pandemic. A prospective national study on prevalence and risk factors. J Affect Disord.

[13] Liu CH, Zhang E, Wong GTF (2020). Factors associated with depression, anxiety, and PTSD symptomatology during the COVID-19 pandemic: clinical implications for U.S. young adult mental health. Psychiatry Res.

[14] Kaur S, Christian H, Cooper MN (2020). Consumption of energy drinks is associated with depression, anxiety, and stress in young adult males: evidence from a longitudinal cohort study. Depress Anxiety.

[15] Hurd LE, Ham LS, Melkonian AJ (2020). Context matters for the socially anxious: moderating role of drinking context on alcohol outcome expectancies. Subst Use Misuse.

[16] Kunas SL, Hilbert K, Yang Y (2020). The modulating impact of cigarette smoking on brain structure in panic disorder: a voxel-based morphometry study. Soc Cogn Affect Neurosci.

[17] Schaumberg K, Wonderlich S, Crosby R (2020). Impulsivity and anxiety-related dimensions in adults with bulimic-spectrum disorders differentially relate to eating disordered behaviors. Eat Behav.

[18] Katsanos AH, de Sa Boasquevisque D, Al-Qarni MA, et al. In-hospital delays for acute stroke treatment delivery during the COVID-19 pandemic*.* Can J Neurol Sci. 2020:1–7. 10.1017/cjn.2020.170 PMC7533482 32741386

[19] Reinstadler SJ, Reindl M, Lechner I. Effect of the COVID-19 Pandemic on treatment delays in patients with ST-segment elevation myocardial infarction*.* 2020; 9. 10.3390/jcm9072183 PMC7408681 32664309

[20] Fullana MA, Hidalgo-Mazzei D, Vieta E (2020). Coping behaviors associated with decreased anxiety and depressive symptoms during the COVID-19 pandemic and lockdown. J Affect Disord.

